# Supplementary material for: Molecular Dynamics Simulations Reveal the Conformational Transition of GH33 Sialidases
Source: Int J Mol Sci. 2023 Apr 6;24(7):6830. doi: 10.3390/ijms24076830 (PMC10095477; doi:10.3390/ijms24076830)
Supplement: Supplementary file 1 [file ijms-24-06830-s001.zip › ijms-2281113-supplementary.pdf]

Supporting information

Molecular Dynamics Simulations Reveal the Conformational Transition of GH33 Sialidases

Xueting Cao [1], Xiao Yang [2], Min Xiao [1,2,*] and Xukai Jiang [1,*]

[1] National Glycoengineering Research Center, Shandong Key Laboratory of Carbohydrate Chemistry and Glycobiology, NMPA Key Laboratory for Quality Research and Evaluation of Carbohydrate-Based Medicine, Shandong University, Qingdao 266237, China

[2] State Key Laboratory of Microbial Technology, Shandong University, Qingdao 266237, China

* Correspondence: minxiao@sdu.edu.cn (M.X.); xukai.jiang@sdu.edu.cn (X.J.)

Running title: Conformational transition of GH33 sialidases

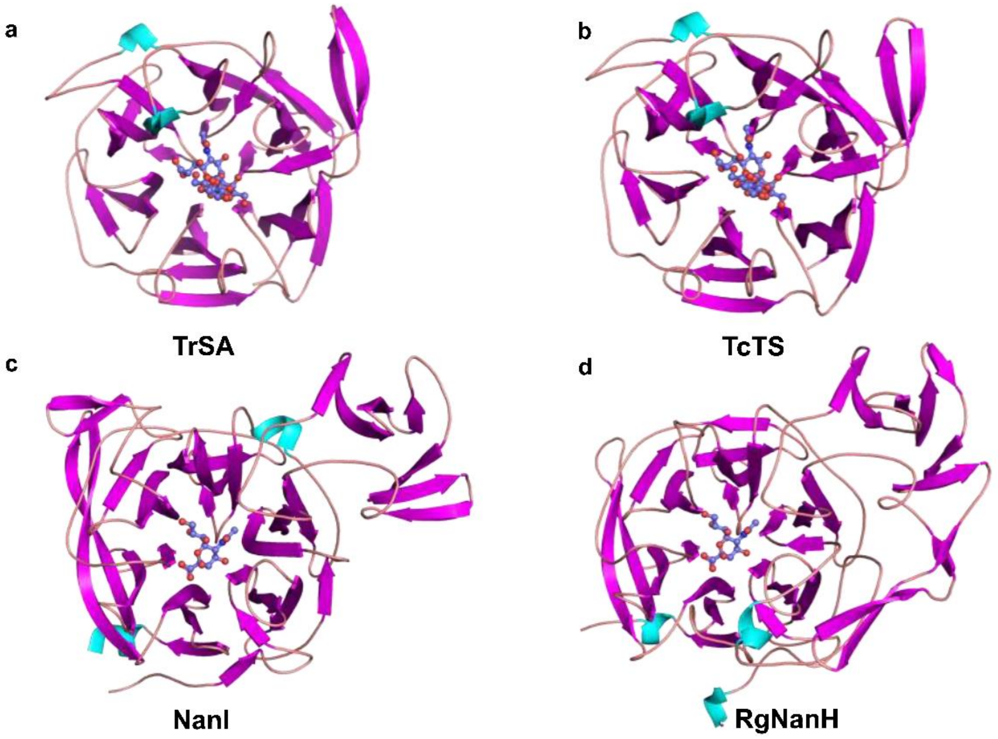

Figure S1. Active site structure of GH33 sialidases. The catalytic domains of GH33 sialidases consisting of a six-bladed β-propeller. The substrates are shown in ball-and-stick models.

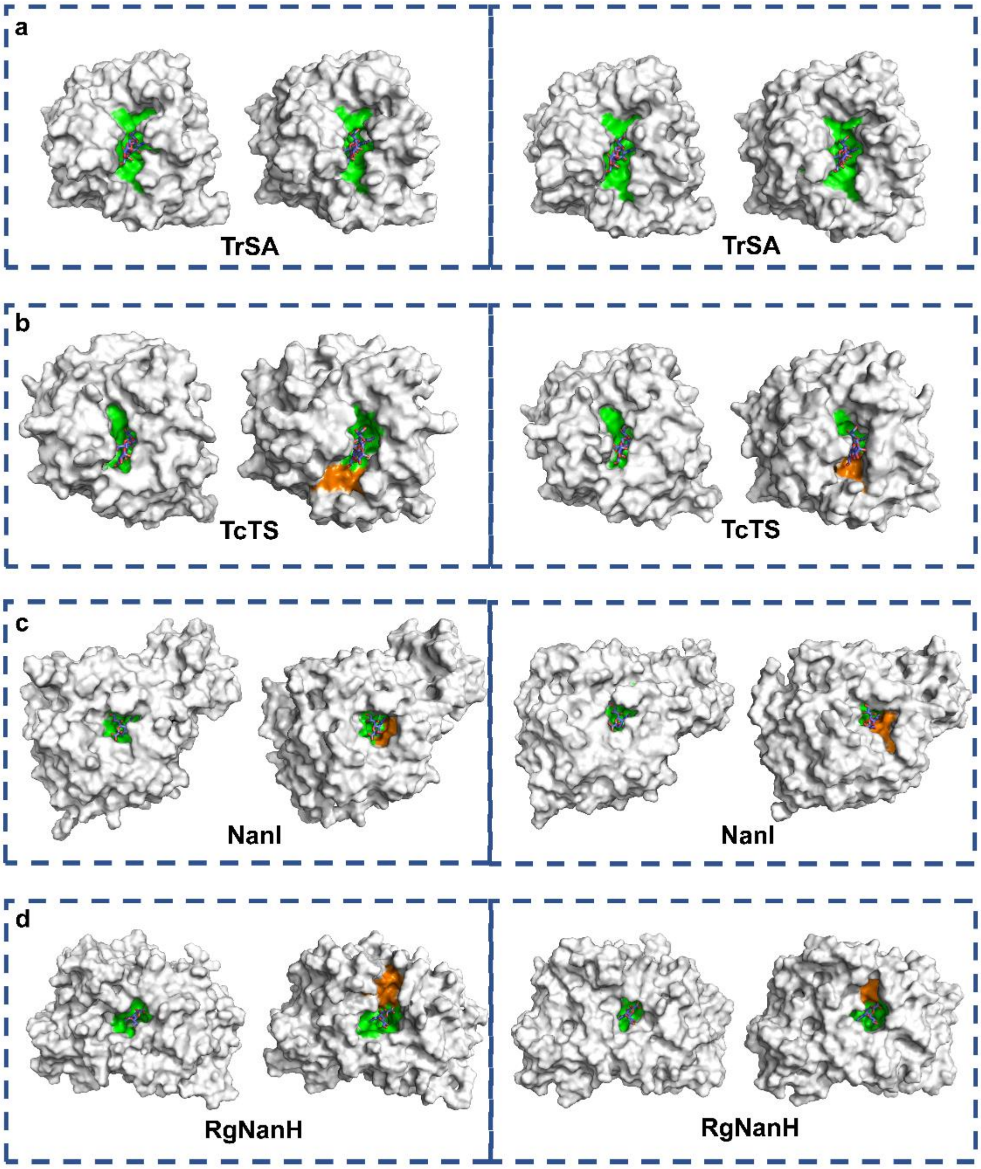

Figure S2. The conformational transition of GH33 sialidases in simulation replicas. The proteins are shown in surface models, the innate active site and the new cleft site of enzyme are shown in green and orange, respectively.

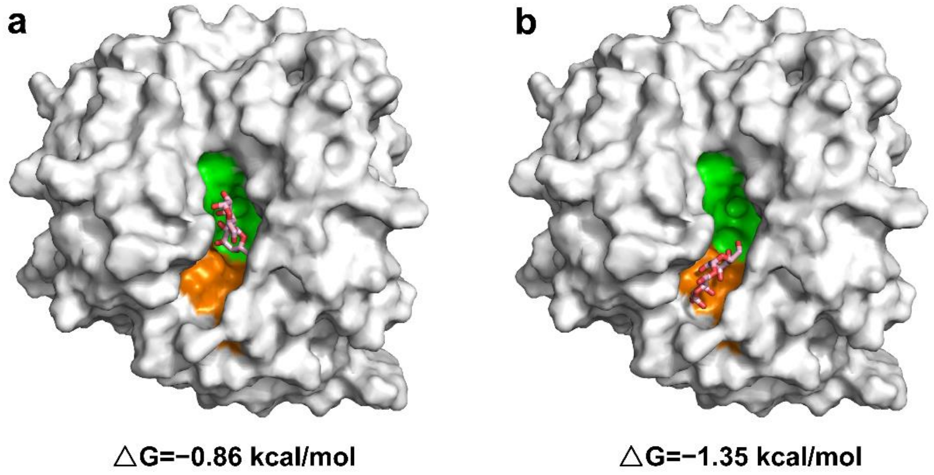

Figure S3. Molecular docking of the TcTS with lactose. TcTS is shown in surface models, the innate active site and the second active site of enzyme are shown in green and orange, respectively. Lactose is shown in pink sticks models.

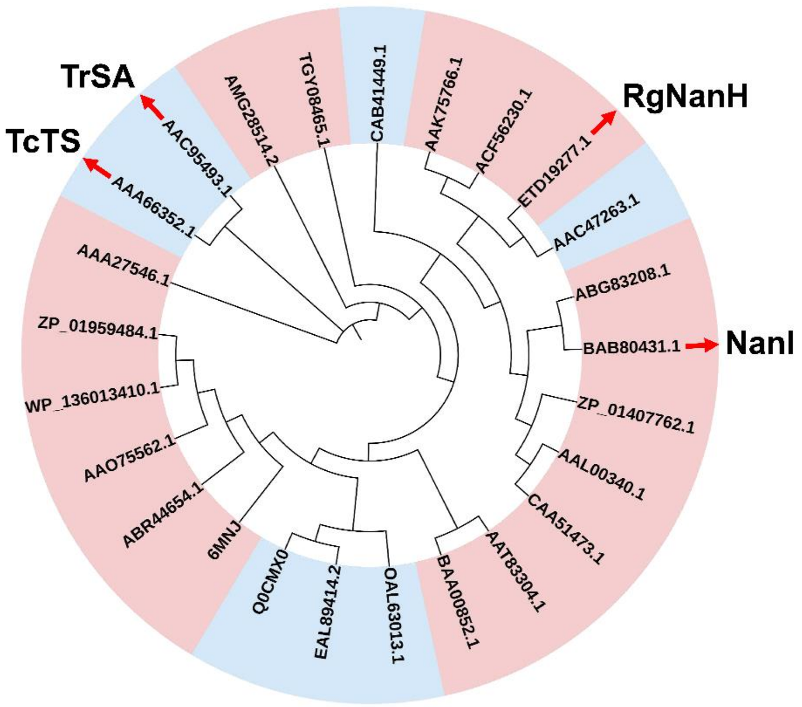

Figure S4. The phylogenetic tree of the GH33 family. Bacteria and eukaryota are highlighted by pink and blue, respectively. The evolutionary locations of four studied sialidases are marked.

Table S1. The residues involved in hydrogen bond interactions with the substrate.

Enzyme-Substrate	Hydrogen bond interactions involved residues							
TrSA-3’-Sialyllactose	36R	54R	60D	97D	246R	315R		
TcTS-3’-Sialyllactose	35R	53R	96D	119Y	120W	230E	245R	314R
NanI-Neu5Ac	266R	285R	291D	328D	493Q	555R	615R	655Y
RgNanH-Neu5Ac	257R	276R	282D	339D	575R	637R	677Y	

Table S2. Key active site residues of sialidases.

Enzyme	catalytic domain	carbohydrate-binding domain	Arg triad	Tyr/Glu	Asp
TrSA	residues 1-375	residues 376-642	36/246/315	343/231	60
TcTS	residues 1-375	residues 376-642	35/245/314	342/230	59
NanI	residues 243-694	residues 1-242	266/555/615	655/539	291
RgNanH	residues 235-723	residues 1-234	257/575/637	677/559	282

Table S3. Detailed information of molecular docking systems.

System	TcTS (innate active site)-Lactose	TcTS (new cleft)-Lactose
Number of rotatable bonds	12	12
Current Total Grid Pts per map	91125	91125
Number of points in X-dimension	44	44
Number of points in Y-dimension	44	44
Number of points in Z-dimension	44	44
X-center	70.801	74.460
Y-center	43.639	50.086
Z-center	54.171	42.692

Data analysis related formulas used in the present study.

RMSD is constructed using the following equation:

$$RMSD = \sqrt{\frac{1}{N}\sum_{i=1}^{N} \delta_i^2}$$

RMSF is constructed using the following equation:

$$RMSF = \sqrt{\frac{1}{T}\sum_{t_j=1}^{T} (x_i(t_j) - \tilde{x}_i)^2}$$

Lennard-Jones interaction energy is constructed using the following equation:

$$V_{LJ}(r_{ij}) = \frac{C_{ij}^{(12)}}{r_{ij}^{12}} - \frac{C_{ij}^{(6)}}{r_{ij}^{6}}$$

Coulomb interaction energy is constructed using the following equation:

$$V_c(r_{ij}) = f\,\frac{q_i q_j}{\varepsilon_r r_{ij}}$$
